# Systematic Analysis of SIN3 Histone Modifying Complex Components During Development

**DOI:** 10.1038/s41598-018-35093-0

**Published:** 2018-11-19

**Authors:** Valerie L. Barnes, Kelly A. Laity, Maksymilian Pilecki, Lori A. Pile

**Affiliations:** Wayne State University, Department of Biological Sciences, Detroit, Michigan 48202 United States of America

## Abstract

Establishment and maintenance of histone acetylation levels are critical for metazoan development and viability. Disruption of the balance between acetylation and deacetylation by treatment with chemical histone deacetylase (HDAC) inhibitors results in loss of cell proliferation, differentiation and/or apoptosis. Histone deacetylation by the SIN3 complex is essential in *Drosophila* and mice, as loss of the scaffolding factor SIN3 or the associated HDAC results in lethality. The objective of this study is to elucidate contributions of SIN3 complex components to these essential processes. We used the *Drosophila* model organism to carry out a systematic functional analysis of the SIN3 complex. We find that SIN3 associated proteins are essential for viability and cell proliferation during development. Additionally, tissue specific reduction of SIN3 complex components results in abnormal wing development. Interestingly, while knockdown of each factor resulted in similar phenotypes, their individual effects on recruitment of SIN3 to polytene chromosomes are distinct. Reduction of some factors leads to large changes in the morphology of the chromosome and/or greatly reduced SIN3 binding. These findings suggest that while individual SIN3 complex components work through distinct molecular mechanisms, they each make a substantial contribution to the overall function of this highly conserved histone deacetylase complex.

## Introduction

The template for eukaryotic transcription is chromatin, comprised of nucleosomes, DNA wrapped around a core of histone proteins. Histones are subject to post-translational chemical modifications, which impact nucleosome structure and protein accessibility to the chromatin template^1^. Histone acetylation is one of the earliest recognized modifications to correlate with transcriptional activity^2^. The enzymes that control acetylation levels, histone lysine acetyltransferases (KATs) and histone deacetylases (HDACs), typically assemble into large protein complexes^3^. Proteins within these enzymatic complexes serve different roles. In some cases, they are critical for recruitment to specific loci. For example, the ING2 subunit of the mammalian SIN3 complex contains a binding domain that recognizes histone H3 trimethylated at lysine 4 (H3K4me3) and is important for recruitment of HDAC1 to target genes in response to DNA damage^4^. In other cases, the complex subunit interacts with a transcription factor allowing for recruitment to specific gene regulatory regions. Mammalian SIN3 was first isolated as a binding partner of the DNA-binding transcriptional repressor Mad^5^.

The histone deacetylase HDAC1, formerly named RPD3 in *Drosophila melanogaster*, is found in multiple multi-subunit protein complexes. These complexes include the nucleosome remodeling (NuRD) complex, corepressor for REST (CoREST) and the SIN3 complex, named for the scaffolding factor SIN3^6^. To add to the complexity, multiple distinct SIN3 complexes are present in a single organism^7^. These SIN3 HDAC complexes vary in subunit isoform specificity and accessory factor composition. Efforts to interrogate the complex components through a set of identical assays to examine the possible roles of individual factors in governing specific biological processes are limited.

SIN3 is a highly conserved protein that is expressed in organisms from yeast to mammals. Mammals have two genes encoding highly similar SIN3 isoforms, SIN3A and SIN3B^5^. *Drosophila* have a single gene, *Sin3A*, which has more sequence similarity to mammalian *SIN3A* compared to *SIN3B*, and from which multiple protein isoforms are produced^8^. Due to the presence of multiple paired amphipathic helical domains, known protein-protein interaction modules, SIN3 is believed to serve as the scaffold for assembly of the SIN3 HDAC complex^9^. While an HDAC was the first histone modifying enzyme found to interact with SIN3^10^, subsequent work from multiple laboratories demonstrated that the histone demethylase KDM5 in mammals, dKDM5/LID in *Drosophila*, interacts with a subset of SIN3 complexes^11^. In studies performed by independent research groups using different organisms, a number of accessory factors including BRMS1, PF1, FAM60A, ING1/2, MRG15, RbAp46/48, RBP1, SAP180, SAP130, SAP30, SAP18 and SDS3 have been reported as members of a SIN3 complex, in addition to the two enzymatic components^12,13^. In an earlier proteomic study to identify isoform-specific SIN3 complexes, we found that the following proteins interact with each of the two major *Drosophila* SIN3 isoforms: HDAC1, CG14220 (SDS3), hat-trick (htk) (ARID4B), CG7379 (ING1/2), Sap130, CG3815 (PF1) and BRMS1^14^. Additionally, we determined that Caf1-55, dKDM5/LID and CG15356 (EMSY) are factors that predominantly interact with the largest and most widely expressed SIN3 isoform, SIN3 220. Apart from the enzymatic activity of HDAC1 and dKDM5/LID, little is understood about the role of the accessory factors in the context of SIN3 complex function.

To address this lack of comprehensive understanding, the objective of this study was to perform a systematic analysis of a defined SIN3 complex in *Drosophila melanogaster*. We sought to identify common and unique phenotypes resulting from reduced expression of complex components, either individually or in combination with reduction of the SIN3 scaffolding factor. We also set out to determine which factors participate in recruitment and/or stabilization of SIN3 onto chromatin, which is believed to be a pre-requisite for complex regulatory activity. Our data indicate that while reduced expression of the majority of the subunits results in highly similar biological phenotypes, only a subset of the factors impact chromatin recruitment. These findings highlight functional specialization of SIN3 complex components.

## Results and Discussion

To examine functions of SIN3 complex subunits, we measured a number of phenotypic traits in flies with reduced levels of SIN3 complex components compared to wild type controls. The complex components investigated included factors previously determined to associate with the largest isoform of SIN3^14^ and are listed in Table 1. To generate flies with reduced protein levels, we used RNA interference (RNAi) to knock down expression of the desired target. Flies carrying a transgene comprised of a UAS regulatory element upstream of a sequence to express double stranded (ds) RNA were crossed to driver flies that express the GAL4 activator. In the progeny of the cross, GAL4 binds the UAS element and activates expression of the dsRNA^15^. This dsRNA is processed by the RNAi machinery and will lead to degradation of the endogenously expressed target mRNA; ultimately resulting in reduced protein expression^16^. For these studies, we utilized a variety of GAL4 driver lines to express GAL4, and thus induce RNAi knock down, in different tissues. For all experiments, we compared the knockdown fly with a control that was the progeny of the same GAL4 driver crossed to either *UAS-GFP*^*RNAi*^ or *UAS-mCherry*^*RNAi*^. These control flies have expression of GAL4 and induction of the RNAi pathway, but no specific *Drosophila* gene is targeted for knock down. As an additional control, to reduce the possibility that observed phenotypes are due to an off-target effect, when available, we utilized more than one RNAi line to target different regions of the targeted RNA. To validate the efficiency of knockdown, we performed RT-qPCR analysis (Supplementary Fig. S1). The assay was performed using RNA isolated from wing imaginal discs, in which only a subset of cells express the dsRNA targeting the gene of interest. The driver for these studies, and for the wing assay described below, is *Ser-Gal4*. This driver contains a portion of the regulatory region for the *serrate (Ser)* gene upstream of the GAL4 coding sequence. *Ser* has a complex expression pattern with prominent expression in the dorsal compartment of the wing imaginal disc^17,18^. We previously determined that this selected driver results in expression of GAL4 in the wing imaginal disc throughout larval development in a pattern that is largely consistent, however that is somewhat broader than, the reported expression of *Ser*^19^. We typically observed RNA levels reduced to about 60-70 % of the level in wild type wing discs in these heterogeneous cell populations.

**Table 1:** SIN3 complex components are required for *Drosophila* viability.

Gene	Stock Name (*UAS-GOI*)	♂ Flies Scored		♀ Flies Scored	
		*Act5C-Gal4/UAS-GOI*	*CyO/UAS-GOI*	*Act5C-Gal4/UAS-GOI*	*CyO/UAS-GOI*
*GFP*	*GFP* ^RNAi^	53	51	61	66
*Sin3A*	*Sin3A* ^RNAi^	0	172	0	189
*HDAC1*	*HDAC1* ^*RNAi-*GD^	0	132	0	144
	*HDAC1* ^*RNAi-*TRiP^	0	158	0	152
*Caf1-55*	*Caf1-55* ^*RNAi*-KK^	0	182	0	162
	*Caf1-55* ^*RNAi*-TRiP^	0	154	0	167
*lid*	*lid* ^*RNAi*-KK^	0	122	0	237
	*lid* ^*RNAi*-TRiP^	0	239	0	334
*htk (ARID4B)*	*htk* ^*RNAi*-TRiP^	0	114	0	111
*Sap130*	*Sap130* ^*RNAi*-GD^	0	126	0	154
*CG15356 (EMSY)*	*CG15356* ^*RNAi*-KK^	0	182	0	199
*CG3815 (PF1)*	*CG3815* ^*RNAi*-TRiP^	0	181	0	188
*CG7379 (ING1/2)*	*CG7379* ^*RNAi*-GD^	0	249	0	237
	*CG7379* ^*RNAi*-KK^	0	167	0	182
*CG14220 (SDS3)*	*CG14220* ^*RNAi*-KK^	0	110	0	175
*Brms1*	*Brms1* ^*RNAi*-KK^	0	108	0	151
	*Brms1* ^*RNAi*-TRiP^	0	99	0	126

The values represent total number of adult flies produced from three independent trials. GOI, gene of interest; KD, knockdown; TRiP, Transgenic RNAi Project at Harvard Medical School; KK, ΦC31 Transgenic RNAi Library from Vienna Drosophila Research Center (VDRC); GD, P-element Transgenic RNAi Library (VDRC).

### SIN3 complex components are essential for viability and wing development

We first tested whether each of the complex components is essential for *Drosophila* viability. We used the *Act5C-Gal4* driver line for ubiquitous expression of GAL4. Progeny of the cross of this driver to the *UAS-target*^*RNAi*^ line results in ubiquitous knockdown of the targeted complex component. Knockdown of each gene coding for a SIN3 complex component resulted in no adult progeny (Table 1). Thus, each tested protein in the complex is encoded by an essential gene. These results are fully consistent and validate previous studies indicating that ubiquitous RNAi knockdown of *Sin3A*, *lid* and *Caf1-55* results in lethality^14,20,21^. Additionally, previous studies indicated that flies carrying homozygous genetic mutations of *HDAC1*^22^ and *Brms1*^23^ are not viable. Data generated in our assay demonstrate that the rest of the genes encoding proteins of the complex, including *htk (ARID4B), Sap130, CG15356 (EMSY), CG3815 (PF1), CG7379 (ING1/2)* and *CG14220 (SDS3)*, are also required for viability.

Because ubiquitous RNAi knockdown resulted in loss of viability, we utilized a conditional knockdown system to further investigate SIN3 complex components. The *Drosophila* wing is a nonessential tissue and has been used by us and others to investigate factors involved in development and cell cycle control^24,25^. We previously determined that SIN3 and dKDM5/LID are necessary for development of a normal wing^19,21^. For this study, we have repeated those experiments to allow for direct comparison with knockdown of the other complex components. As in those prior studies, here we used the *Ser-Gal4* driver to induce RNAi knockdown of the SIN3 complex components in wing imaginal discs and observed the resulting adult wing morphology.

**Figure 1:**
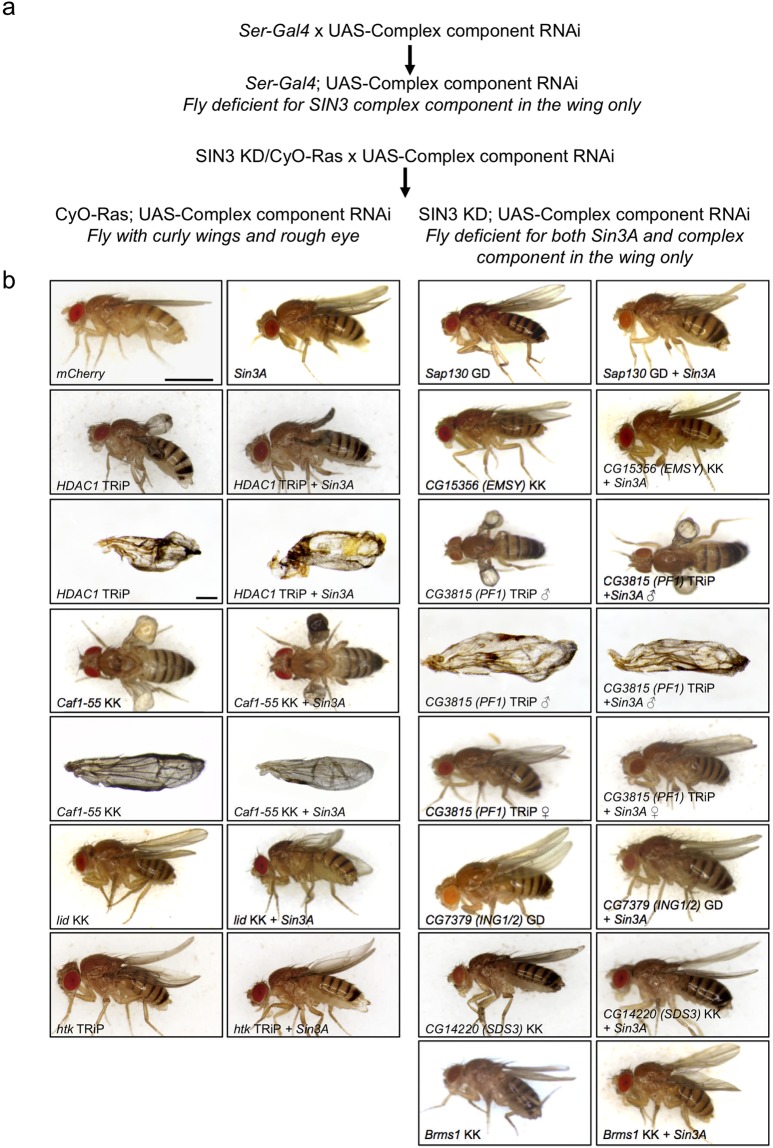
SIN3 complex components are necessary for normal wing development. (**a**) *Ser-Gal4* or SIN3 KD/CyO-Ras (Serrate-Gal4 - > UAS-SIN3 RNAi, which is balanced over chromosome II balancer CyO-Ras, marked by a curly wing and a rough eye phenotype) are crossed to each of the complex component RNAi lines, as well as to mCherry RNAi, as a control. Progeny are scored for wing phenotype caused by single or double knockdown as shown. (**b**) Micrographs of flies or wings with either individual knockdown (left panels) or with the gene of interest along with *Sin3A* knockdown (right panels). *mCherry*: control flies expressing *mCherry* dsRNA. Scale bar (whole flies) 1 mm. Scale bar (wings) 100 μm.

We first tested whether the individual complex component was necessary for wing development. Individual reduction of HDAC1, Caf1-55 or CG3815 (PF1) resulted in severely malformed wing phenotypes (Fig. 1, Table 2, Supplementary Fig. S2). The wing tissue is blistered and blackened. This phenotype is more severe compared to that resulting from knockdown of either *Sin3A* or *lid*. HDAC1 and Caf1-55 are each found in additional complexes apart from SIN3. HDAC1 is a component of other HDAC complexes including NuRD and CoREST^6^. Caf1-55 associates not only with HDACs, but also KATs and chromatin assembly factors^26^. It is possible that the loss of each of these factors impacts the function of not only the SIN3 complex but also the other chromatin organization complexes in which these factors are found. The severe disruption of wing morphology is thus likely due to effects on multiple developmental pathways. The other factor found to yield the blistered phenotype upon knockdown is CG3815 (PF1). Little is known about PF1 in flies and work in other organisms has focused on the role of this factor as a component of the SIN3 complex. In triple negative breast cancer cells, disruption of the interaction between PF1 and SIN3A altered the expression of cancer-related genes and importantly, decreased the metastatic potential of cancer cells in a mouse tumor model^27^. These authors additionally found that knockdown of PF1 phenocopies the effects of disruption of the PF1 and SIN3A interaction, indicating that a main role for PF1 is as part of the SIN3 complex. The data from the fly wing development assay suggests that, in flies, PF1 might function apart from SIN3. Interestingly, the blistered wings are only found in the male *CG3815 (PF1)* knockdown flies. The wings of the female *CG3815 (PF1)* knockdown flies are curved, similar to those of the *Sin3A* knockdown flies (Fig. 1). These data, as well as a few additional sex specific discrepancies in observed phenotypes as noted below, suggest that when compared to females, males are more sensitive to a reduction in SIN3 complex activity. We observed that the rest of the tested SIN3 complex subunits, including htk (ARID4B), Sap130, CG15356 (EMSY), CG7379 (ING1/2), CG14220 (SDS3) and BRMS1, are necessary for formation of a normal straight adult wing (Fig. 1, Table 2). For these flies, besides the curvature of the wing, no additional wing defects were noted upon visual inspection. While the majority of adult *CG14220* (*SDS3*) and *Brms1* knockdown female flies had curved wings, some had straight wings supporting the idea that females are less sensitive to reduced SIN3 complex function as compared to males. Like wild type strains, progeny of the control cross had straight wings. When multiple RNAi lines were tested for an individual gene, the resulting phenotypes were similar across the lines. These results suggest the phenotypes are not due to off-target effects but rather are due to reduced expression of the targeted gene.

**Table 2:** SIN3 complex components are essential for wing development.

Stock Name (UAS-GOI)	*Ser-Gal4/UAS-GOI*				SIN3 KD*/UAS-GOI*			
	♂		♀		♂		♀	
	# Scored	% with phenotype	# Scored	% with phenotype	# Scored	% with phenotype	# Scored	% with phenotype
*HDAC1* ^*RNAi-*GD^	97	100	116	100	92	100	115	100
*HDAC1* ^*RNAi-*TRiP^	192	93	213	76	176	94	226	94
*Caf1-55* ^*RNAi*-KK^	50	100	75	100	19	100	90	100
*Caf1-55* ^*RNAi*-TRiP^	156	100^*^	216	95	69	100	140	100
*lid* ^*RNAi*-KK^	87	100	127	100	101	100	136	100
*lid* ^*RNAi*-TRiP^	120	100	149	100	51	100	207	100
*htk* ^*RNAi*-TRiP^	146	100	204	87	189	100	242	79
*Sap130* ^*RNAi*-GD^	160	100	197	98	196	100	266	100
*CG15356* ^*RNAi*-KK^	180	100	155	100	177	100	176	100
*CG3815* ^*RNAi*-TRiP^	187	100	87	100	108	100	81	100
*CG7379* ^*RNAi*-GD^	212	100	245	100	222	100	203	100
*CG7379* ^*RNAi*-KK^	170	93	182	97	189	100	159	100
*CG14220* ^*RNAi*-KK^	100	100	202	60	114	100	138	100
*Brms1* ^*RNAi*-KK^	263	78	478	55	145	100	162	100
*Brms1* ^*RNAi*-TRiP^	180	100	110	100	92	100	117	100

Results are from at least three independent biological replicates. For specific phenotypes, refer to Fig. 1. ^*^57% with blistered wings, 43% were curved.

We note that sex specific phenotypic differences comparing flies with mutation or reduced expression of SIN3 complex components to wild type have been reported previously. For example, we previously observed that overexpression of dKDM5/LID in the context of *Sin3A* wing specific knockdown suppresses the curved wing phenotype in female flies by not in males^21^. Additionally, male flies that carry a mutation in *lid* have shorter life spans and a higher sensitivity to the presence of paraquat-induced reactive oxygen species as compared to the females with the mutation^28^. Mutations in *HDAC1* have also been found to yield sex specific phenotypic variation. Flies carrying certain specific alleles of *HDAC1* survive at significantly lower rates as compared to their genetically identical females counterparts^29^. We do not have a definitive answer as to the mechanism controlling the sex specific differential sensitivity to mutation in SIN3 complex components. It is possible that the differences are related to the epigenetic variation linked to the presence of the heterochromatic Y chromosome in male flies and/or the single X chromosome, which is subject to dosage compensation controlled in part by histone acetylation^30^. In addition to the possible impact of dosage compensation mechanisms, male and female flies have differences in their genome-wide chromatin landscape^31^. It is possible that the SIN3 histone modifying complex controls some of these sex specific differences, which then differentially affect genes important for normal wing morphology.

An additional observation is that although we determined efficiency of knockdown in the wing imaginal discs at approximately 60 and 70% of control levels (Supplementary Fig. S1), to our knowledge, no wing abnormalities have been observed in the flies heterozygous for genetic mutations, and thus with perhaps 50% reduction, in the SIN3 complex factor. As noted above, the *GAL4* expression pattern controlled by the *Ser-Gal4* driver is complex and thus the amount of knockdown is likely variable across the wing imaginal discs cells, with some cells affected very little and others a lot.

We next examined the effect of reducing levels of SIN3 along with a second component of the complex. We previously determined that dual knockdown of *Sin3A* and *lid* resulted in a more severe phenotype compared to knockdown of either factor alone^21^. For the current study, we used a fly line with constitutive knockdown of *Sin3A* in the wing imaginal disc, *SIN3 KD I*, previously generated in our laboratory^19^. This line carries both the *Ser-Gal4* and *UAS-SIN3*^*RNAi*^ transgenes on a single chromosome. We crossed these flies with the *UAS-target*^*RNAi*^ lines for the SIN3 complex components and observed wing morphology in adult progeny (Fig. 1, Table 2, Supplementary Fig. S2). The wing phenotype in the double knockdown flies was equivalent to the phenotype recorded for individual knockdown. For example, no change to the curved wing phenotype observed in the *htk (ARID4B)* knockdown was observed when *htk (ARID4B)* and *Sin3A* were simultaneously reduced by RNAi. These data suggest that the majority of the factors work through a similar mechanism to impact the same pathways for wing development. Reduction of HDAC1, CAF1-55 and CG3815 (PF1) leads to flies with a more severe phenotype compared to the *Sin3A* knockdown curved wing, which is not modified when combined with SIN3 reduction. This finding suggests that those three factors have function outside of the SIN3 complex. As noted, HDAC1 and CAF1-55 are part of multiple non-SIN3 containing chromatin modification complexes^6,26^. The number of male flies for the *Caf1-55*^*KK*^*, Sin3A* double knockdown genotype is low, possibly due to lethality in the pupal stage as we observed pupal cases from which flies did not eclose. We do not have an explanation as to reason why the wing specific knockdown would result in male lethality. Taken together, the results indicate all tested components of the SIN3 complex are required for normal wild type development of the *Drosophila* wing. Because wing specific RNAi of each factor results in a similar curved wing phenotype and they were found to biochemically associate in a previously conducted co-immunoprecipitation experiment^14^, it is possible that most of the components work as part of a single complex. Because of the distinct phenotypes observed in the wing specific RNAi knockdown flies, HDAC1, CAF1-55 and CG3815 (PF1) likely have some activity outside of their roles as members of the SIN3 complex. Whether other complex factors work outside of their association with SIN3 cannot be ruled out and future work identifying possible non-SIN3 containing additional complexes will shed light on this possibility.

### Components of the SIN3 complex are necessary for cell proliferation or cell viability during development

**Figure 2:**
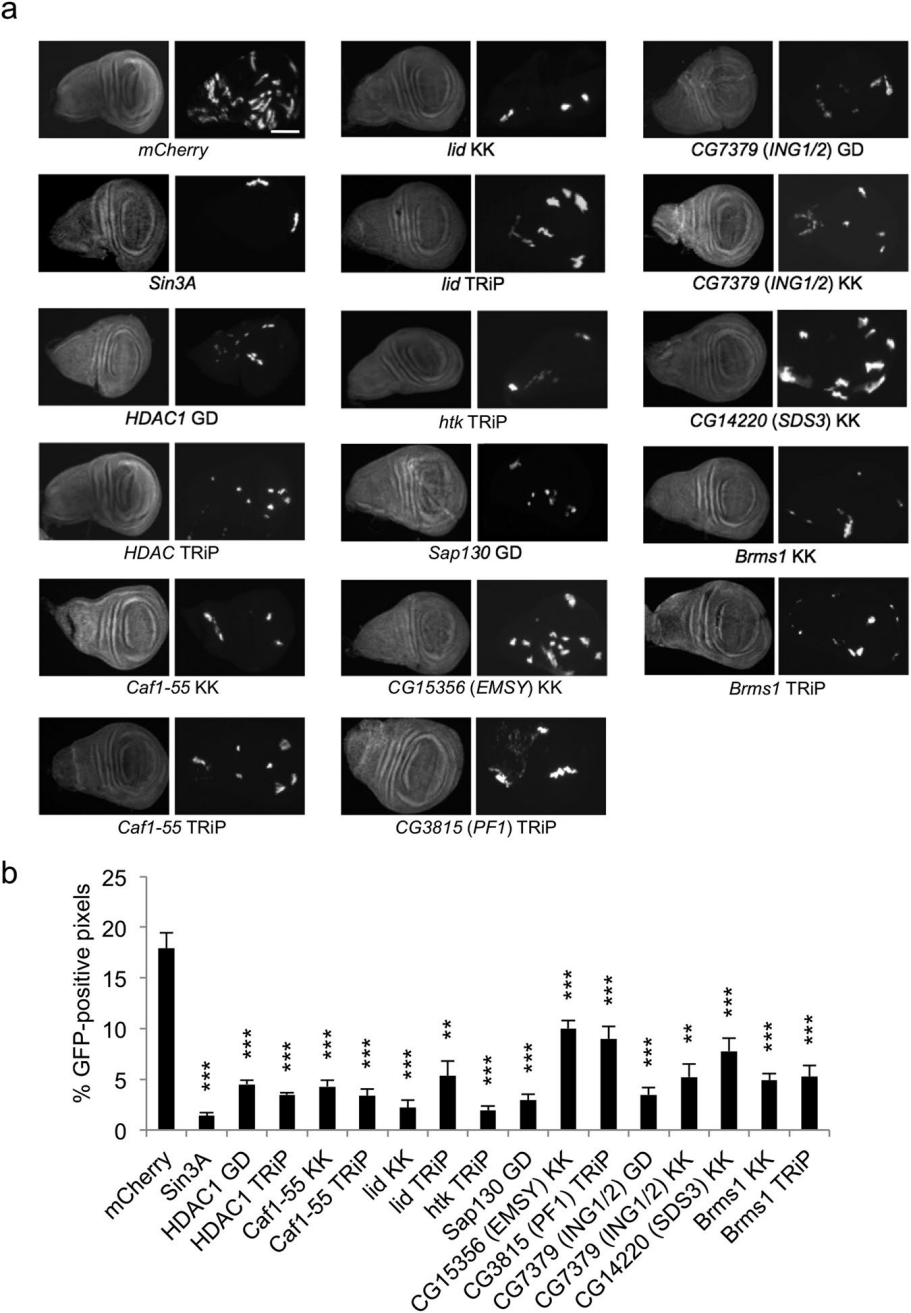
Knockdown of SIN3 complex components affects proliferation of wing imaginal disc cells. (**a**) *mCherry* RNAi control and knockdown wing disc clones were generated using the flip-out GAL4 system and immunostained with antibody to GFP. DAPI staining is in the left panel of paired images for each fly line. GFP signal is shown in the right panel. Scale bar 100 μm. (**b**) Quantification of GFP signal in wing imaginal discs. Results are the average of GFP positive pixel counts from three biological replicates with at least 17 wing imaginal discs in total for each sample. Error bars represent standard error of the mean. ^**^P < 0.01, ^***^P < 0.001.

Early studies indicated that SIN3 and some complex components, including HDAC1 and CAF1-55, are required for proliferation of the *Drosophila* S2 cultured cell line^32^. It was subsequently determined that SIN3, HDAC1, the histone demethylase dKDM5/LID and CAF1-55 are also critical for cell proliferation and cell survival during *Drosophila* development^17,19,21,33–35^. To investigate if other components of the complex are necessary for cell proliferation during development, we performed clonal analysis in wing imaginal disc cells. For these studies, we utilized the heat shock “flip-out” GAL4 driver (Actin > GAL4) to activate expression of a UAS-linked RNAi target along with a GFP marker in random clones of cells via the heat-shock induction of FLP recombinase at a precise time point in larval development. Once activated, cell clones will continue to have reduced target gene expression and the effect of this reduced expression on cell proliferation can be measured by the size and number of the GFP positive cells. Reduction of all SIN3 complex components resulted in fewer GFP positive cells. In some instances, the clones are smaller in size and for others, the clones are fewer in number, relative to the clones produced in the mCherry RNAi controls (Fig. 2). The low yield of GFP positive cells in discs with reduced expression of a complex component is consistent with slower growth and progression through the cell cycle as compared to wild type cells. It is also possible that cells with RNAi reduced expression of a particular factor undergo apoptosis. In our previous studies with RNAi knockdown of *Sin3A*, *HDAC1*, *Caf1-55* and *lid* in S2 cultured cells, however, we did not observe an increase in the number of dead cells in the population following treatment with the dsRNA to target the individual gene^21,32^.

The human homologs of a number of SIN3 complex factors, including ARID4B, EMSY, PF1, ING1/2 and BRMS1, are known to function in cell proliferation and the level of their expression has been linked to growth of cancer cells. Elevated expression of ARID4B was found to promote metastasis in a mouse model of human breast cancer^36^. EMSY was first identified in a screen to look for factors associated with BRCA2 and breast cancer^37^. In more recent work, researchers analyzed RNA-seq data available in the TCGA Research Network and found that EMSY, along with the demethylase KDM5A as well as SIN3B, is overexpressed in primary tumors^38^. As noted above, disruption of the interaction between PF1 and SIN3B limits the metastatic potential of breast cancer cells in a mouse tumor model^27^. ING1 and ING2 are members of a family of proteins involved in cell proliferation^39^. In flies, the single gene *CG7379* is approximately 40% similar to both human ING1 and ING2 (Supplementary Fig. S3). ING1 was first isolated in a screen to discover novel tumor suppressor genes^40^. ING proteins are generally thought to be tumor suppressors, but have been found to be overexpressed in some cancer types^41^. BRMS1 was identified in a screen for factors important for suppression of human breast cancer carcinoma metastasis and subsequently found to serve a similar role in other tumor types^42,43^. It is interesting to note that some SIN3 complex factors, including SIN3, HDAC1, KDM5B, ARID4B, EMSY and PF1, are generally required for cell proliferation, while expression of ING2 and BRMS1 limits growth of many cancer cell types. Data generated from the clonal analysis in *Drosophila* wing imaginal discs indicates that the cell proliferation function of these factors, and indeed of the SIN3 complex, is conserved. RNAi knockdown of *CG7379* (*ING1/2*) and *Brms1* led to reduced cell proliferation in the developing wing tissue. This finding suggests that their function in these cells is similar to that of other complex components; they are important for cell proliferation rather than functioning as tumor suppressors.

### Multiple components in the SIN3 complex can recruit SIN3

SIN3 is described as corepressor as it does not contain a DNA-binding domain for gene targeting. To recruit the complex to promoters where it functions to regulate gene expression, sequence-specific DNA-binding transcription factors interact with SIN3 and/or components of the complex^9^. We asked if any of the complex components are critical for recruitment of SIN3 to chromatin. For this analysis, we used polytene chromosomes prepared from *Drosophila* larval salivary glands. We utilized the *feb36-GAL4* driver line to induce RNAi and reduce the level of the SIN3 complex factor in salivary glands^44,45^. Chromosome preparations from third instar larval salivary glands were probed with antibody to SIN3 and also stained with DAPI to visualize the DNA.

**Figure 3:**
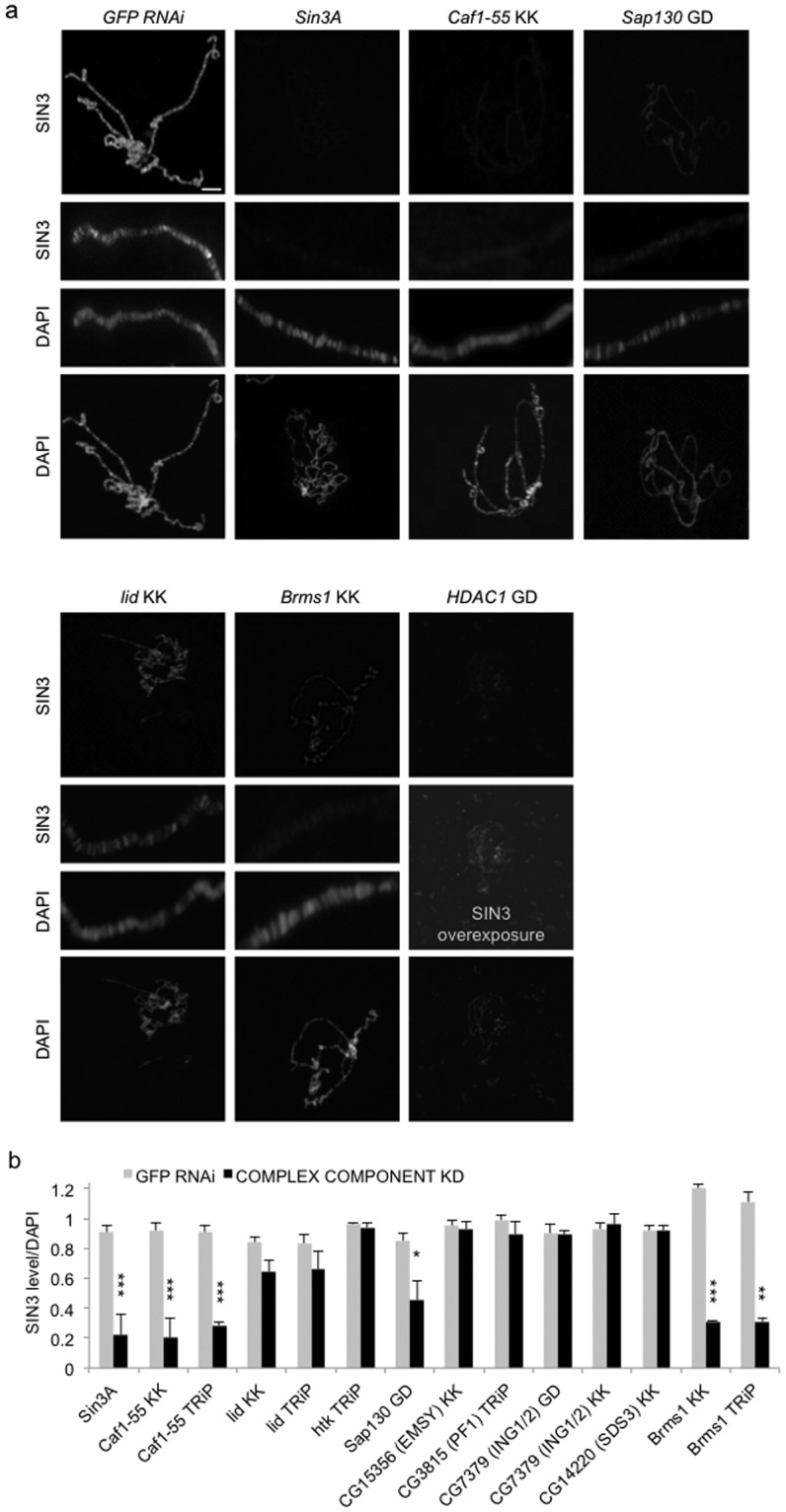
Control of SIN3 chromatin recruitment by SIN3 complex factors. (**a**) Polytene chromosome spreads were prepared from salivary glands of *GFP* RNAi control and knockdown flies. Chromosomes were immunostained with antibody to SIN3 and counterstained with DAPI. Images of higher magnification (200x original image) of individual arms are inset between full genome SIN3 and DAPI images. Scale bar 10 μm. (**b**) Quantification of SIN3 staining. Results are the average of three to five biological replicates including at least 19 chromosomes in total for each sample. Error bars represent standard error of the mean. ^*^P < 0.05, ^**^P < 0.01, ^***^P < 0.001.

Unlike with the assays described above, knockdown of individual SIN3 complex factors resulted in different phenotypic effects. Knockdown of only a limited set of factors impacted SIN3 binding. Additionally, overall chromatin structure was affected by knockdown of some complex components and not others (Fig. 3). First, RNAi of *HDAC1* led to disruption of polytene chromatin structure. The salivary glands were smaller and overall DAPI staining of nuclei was low compared to wild type controls. The chromosomes lack integrity; the banding pattern is disrupted and less distinct as compared to control chromosomes (Fig. 3). SIN3 binding is apparent along the chromosome arms, but due to the disrupted morphology, we were unable to quantify the level of staining. We conclude from these data that HDAC1 is important for formation or stability of normal structure of the polytene chromosome but may not be essential for recruitment of SIN3. Knockdown of three factors, *Caf1-55*, *Brms1*, and *Sap130*, resulted in strongly diminished SIN3 recruitment (Fig. 3). The effect of reduction of dKDM5/LID led to a more subtle effect on SIN3 staining levels. The reduction of overall SIN3 binding was apparent, but not statistically significant (Fig. 3). Other tested components of the complex, including htk (ARID4B), CG15356 (EMSY), CG3815 (PF1), CG7379 (ING1/2) and CG14220 (SDS3), are not critical for SIN3 chromosomal recruitment as no effect on SIN3 binding was observed along polytene chromosomes prepared from salivary glands with reduced levels of these factors (Fig. 3 and Supplementary Fig. S4).

It is interesting to note that reduction of multiple individual factors of the complex led to such a strong effect on SIN3 recruitment. Perhaps Caf1-55, BRMS1 and Sap130 form a sub-complex. One possibility is that loss of one those three factors affects assembly of the other two of the three into the sub-complex. This assembled sub-module may be key for recruitment of SIN3 and the other components of the complex. Caf1-55 has been demonstrated to interact with histones^46^ and this interaction may be critical for either recruitment or stability of SIN3 to chromatin. The data support the idea that certain factors are important for SIN3 chromatin recruitment. The lack of staining on polytenes, however, could possibly be because the chromatin landscape itself is altered such that SIN3 or the complex is prevented from binding. For example, loss of a factor could lead to binding of an activator that prevents a transcription factor involved in recruiting SIN3 from binding. Future work will be necessary to examine the mechanism of how various SIN3 complex components affect SIN3 chromatin binding.

## Summary

In this study, we have analyzed the contributions of components of the SIN3 complex to key developmental processes including viability, tissue development and cell proliferation. We found that all tested complex components are essential for viability and critical for cell proliferation and development of adult tissue. Unlike these shared requirements, only a limited set of the factors is necessary for chromatin recruitment of the SIN3 scaffolding component. Reduction of each Caf1-55, BRMS1 and Sap130 largely diminishes the binding of SIN3 to chromosomes compared to wild type. Further work will be necessary to dissect protein-protein interactions of the complex to determine how loss of some factors and not others impacts SIN3 binding. Additionally, we note that while loss of only a few factors affects SIN3 chromatin binding, all work in similar biological processes necessary for cell proliferation and development to adulthood. The complex thus likely works as an integrated module to regulate gene activity, with each component making a substantial contribution to function.

## Methods

### Drosophila stocks

*Drosophila melanogaster* stocks were maintained at 25 °C, and crosses were performed at 27 °C, according to standard laboratory procedures. The stocks that were used can be found in Table 3. SIN3 KD (knockdown) flies were created by crossing *w*^1118^; *Ser-Gal4/UAS-SIN3PanKD* females to CyO-Ras/Sco males. Recombinant progeny were selected based on their eye color and verified by crossing to *w*^1118^ flies and observing the curved wing phenotype characteristic of SIN3 KD in the wing tissue^19^. *hsFLP; Act5c* > *CD2* > *Gal4, UAS-EGFP* were a gift from the Bohmann lab at University of Rochester.

**Table 3:** Fly stocks used.

Stock number	Stock Name	Genotype
BDSC 6791	*Ser-Gal4*	w[*]; P{w[+mC] = Ser-GAL4.GF}1 P{Ser-GAL4.GF}2
BDSC 4414	*Actin-Gal4*	y[1] w[*]; P{w[+mC] = Act5C-GAL4}25FO1/CyO, y[+]
BDSC 29968	*Feb36-Gal4*	w[*]; P{w[+mW.hs] = GawB}Feb36
Bohmann Lab Transgenic	*hsFLP-Gal4*	*hsFLP; Act5c* > *CD2* > *Gal4, UAS-EGFP*
Pile Lab Transgenic	SIN3 KD	w1118; Ser- > SIN3PanKD/CyO-Ras
BDSC 9331	*GFP* ^RNAi^	w[1118]; P{w[+mC] = UAS-Avic\GFP.dsRNA.R}143
BDSC 35785	*mCherry* ^*RNAi-*TRiP^	y[1] sc[*] v[1]; P{y[+t7.7] v[+t1.8] = VALIUM20-mCherry}attP2
Pile Lab Transgenic	*Sin3A* ^RNAi^	w1118;pWiz:SIN3PanKD
VDRC 46930^**^	*HDAC1* ^*RNAi-*GD^	w[1118]; P{GD17233}v46930/TM6,Tb
BDSC 31616	*HDAC1* ^*RNAi-*TRiP^	y[1] v[1]; P{y[+t7.7] v[+t1.8] = TRiP.JF01401}attP2
VDRC 105838	*Caf1-55* ^*RNAi*-KK^	P{KK102930}VIE-260B
BDSC 31714	*Caf1-55* ^*RNAi*-TRiP^	y[1] v[1]; P{y[+t7.7] v[+t1.8] = TRiP.HM04021}attP2
VDRC 103830	*lid* ^*RNAi*-KK^	P{KK102745}VIE-260B
BDSC 28944	*lid* ^*RNAi*-TRiP^	y[1] v[1]; P{y[+t7.7] v[+t1.8] = TRiP.HM05155}attP2
BDSC 31754	*htk* ^*RNAi*-TRiP^	y[1] v[1]; P{y[+t7.7] v[+t1.8] = TRiP.HM04064}attP2
VDRC 31394	*Sap130* ^*RNAi*-GD^	w[1118]; P{GD7168}v31394
VDRC 106820	*CG15356* ^*RNAi*-KK^	P{KK107847}VIE-260B
BDSC 56908	*CG3815* ^*RNAi*-TRiP^	y[1] sc[*] v[1]; P{y[+t7.7] v[+t1.8] = TRiP.HMS04327}attP40
VDRC 27988	*CG7379* ^*RNAi*-GD^	w[1118]; P{GD12222}v27988
VDRC 103928	*CG7379* ^*RNAi*-KK^	P{KK102827}VIE-260B
VDRC 105162	*CG14220* ^*RNAi*-KK^	P{KK102695}VIE-260B
VDRC 105494	*Brms1* ^*RNAi*-KK^	P{KK108153}VIE-260B
BDSC 42533	*Brms1* ^*RNAi*-TRiP^	y[1] v[1]; P{y[+t7.7] v[+t1.8] = TRiP.HMJ02100}attP40

^**^Original VDRC stock is balanced over TM3, Sb; we rebalanced over TM6, Tb for scoring 3^rd^ instar larvae for wing disc dissection.

### Reverse transcription PCR assay

Total RNA was extracted from 20 to 30 wing discs isolated from wandering third instar larvae using the RNeasy mini kit (Qiagen). cDNA was generated from total RNA using the ImProm-II Reverse Transcription System (Promega) with random hexamers. The cDNA was used as template in a quantitative real-time PCR (qPCR) assay. The analysis was performed using ABsolute SYBR Green ROX master mix (Fisher Scientific) and carried out in a Stratagene Mx3005P real-time thermocycler. Primers used for analysis are given in Supplemental Table S1. *Taf1* and *Pgk* were used to normalize cDNA amounts in the 2^−ΔΔCt^ comparative analysis^47^.

### Imaging flies

Whole flies were imaged at 30x magnification using an Olympus DP72 camera coupled to an Olympus SZX16 microscope. Wings were imaged at 80x magnification using a SPOT RT color camera coupled to a Leica MZ125 microscope.

### Clonal analysis

hsFLP;Act5C > CD2 > Gal4,UAS-EGFP virgin females were crossed to *mCherry*^*RNAi-TRiP*^ males or *UAS-SIN3* complex component RNAi to generate random GFP positive clones via the heat shock flip-out system. Embryos were collected for four hours and then after 48–52 hours, 2^nd^ instar larvae were subjected to heat shock at 37 °C for two hours. After returning to 27 °C, wandering 3^rd^ instar larvae were dissected and immunostained 120 hours after egg laying^19^. Antibody against GFP (1:1000; Abcam, ab1218) followed by sheep anti-mouse Alexa 488 (1:2000; Life Technologies, A11001) was used for staining. Visualization and imaging was done using an Olympus BX53 compound microscope with a DP72, 12.8 megapixel camera. Images were processed using Olympus CellSens software. Clones were analyzed in a minimum of 20 discs per genotype using Photoshop CS to count the GFP positive pixels for each immunostained disc and comparing that number to the total number of pixels in the DAPI-stained disc^48^.

### Polytene chromosome staining

Polytene chromosome preparation and staining methods were modified from the protocol outlined here^49^. A primary antibody against SIN3 (1:1000)^50^ was followed by a secondary antibody Alexa Fluor 594 (1:400) (Life Technologies). Chromosome spreads were prepared from a minimum of three independent parental crosses and representative images are shown. A minimum of four slides of each control and experimental genotype were prepared at the same time and photographed using identical exposure times. Chromosomes were imaged using an Olympus BX53 compound microscope with a DP72, 12.8 megapixel camera at 400x. Five to ten chromosomal spreads were chosen from each slide for imaging, and images were processed identically using Olympus CellSens software. To quantify SIN3 immunofluorescence signals on polytene chromosomes relative to DAPI intensity, we used a program developed in Matlab 7.4.0 from the protocol outlined here^51^. Briefly, batches of control and experimental images, processed identically, are input into the program, which uses two fluorescent channels (DAPI and Alexa Fluor 594). The program applies a mask to each chromosome, removing non-chromosomal antibody staining from the pixel-based quantification.

### Statistical analyses

All significance values were calculated by the two sample Student’s *t* test using GRAPHPAD software. http://www.graphpad.com/quickcalcs/index.cfm.

## Electronic supplementary material

Supplementary Information

## Data Availability

All data generated or analyzed during this study are included in this published article and its Supplementary Information files.
