# Supplementary material for: Systematic Analysis of SIN3 Histone Modifying Complex Components During Development
Source: Sci Rep. 2018 Nov 19;8:17048. doi: 10.1038/s41598-018-35093-0 (PMC6242963; doi:10.1038/s41598-018-35093-0)
Supplement: Supplementary file 1 — Supplementary Information [file 41598_2018_35093_MOESM1_ESM.pdf]

Supplementary Information for:

Systematic Analysis of SIN3 Histone Modifying Complex Components During Development

Valerie L. Barnes, Kelly A. Laity, Maksymilian Pilecki, and Lori A. Pile

Wayne State University, Department of Biological Sciences, Detroit, Michigan, 48202, United States of America

Correspondence and requests for materials should be addressed to L.A.P. (email:loripile@wayne.edu)

Table S1: Primers used for RT-qPCR analysis

Gene	Primer orientation	Primer sequence (oriented 5' to 3')
Taf1	Forward	CTGGTCCTGGTGAGGTGA
	Reverse	CCGGATTCTGGGATTTGA
Pgk	Forward	CCCCCCGGTGTCTTTGAG
	Reverse	GCCGTCCATGATGGACTTG
HDAC1	Forward	AACAGCAACAAGGCATCCTC
	Reverse	TATTAGACGCCGTCGATTCC
Caf1-55	Forward	GCCTGTGTAATCGCCACC
	Reverse	CTTGCTGGGATGCTTCGT
lid	Forward	TCGTGCGAAAAGACACAGAA
	Reverse	GCCCGATCTGCTTCACCAGC
htk	Forward	CGGGAGAGGTCAATCCCT
	Reverse	TCGAGCCCTGCATTCTTC
Sap130	Forward	AAGCTCGGGCTGTGTTTG
	Reverse	CGTTATTGGAGCATCCCG
CG15356 (EMSY)	Forward	CAAAGCCTGCGAGGAAAA
	Reverse	CCACGCGGATATTTCAGG
CG3815 (PF1)	Forward	CTGTATGCCTGCGACGTG
	Reverse	GACGCGCTTCTTCAGCTC
CG7379 (ING1/2)	Forward	GGTTCCATTTTTCGTGCG
	Reverse	TCTTTGAGGAACTGCGCC
CG14220 (SDS3)	Forward	CAACAACATGGGCACCAA
	Reverse	GGCCCAGTTCCTCCATCT
Brms1	Forward	GCTTGGAGCGCCAATTTA
	Reverse	TTTGCGGCTGGACAAACT

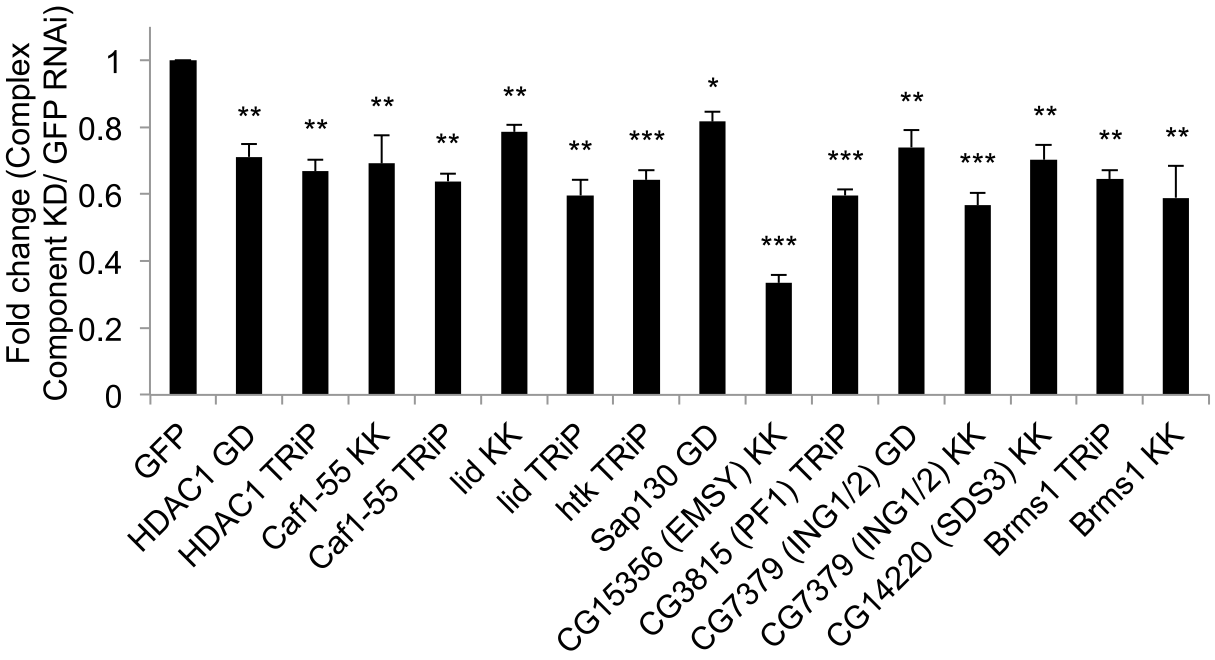

Fig. S1. The *Ser-Gal4* driver can be used to effectively knock down expression of SIN3 complex components in larval wing discs. RT-qPCR analysis was performed on RNA isolated from wing discs containing *Ser-Gal4* and *UAS-GOI^{RNAi}* transgenes. Relative levels of gene expression are indicated. Error bars represent standard error of the mean. (*)P<0.05, (**)P<0.01, (***)P<0.001.

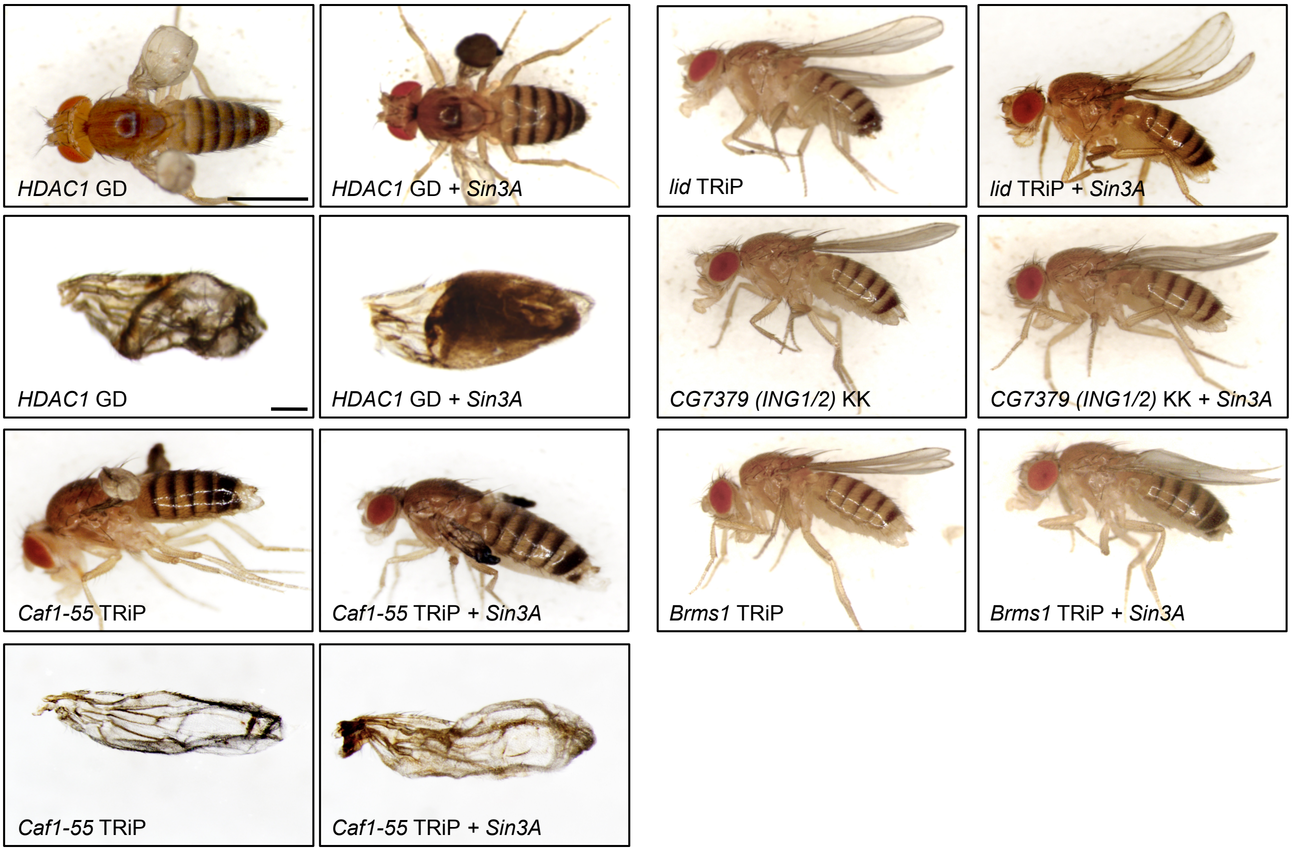

Fig. S2. SIN3 complex components are necessary for normal wing development: Additional stocks tested. Micrographs of flies or wings with either individual knockdown (left panels) or with the gene of interest along with *Sin3A* knockdown (right panels). Scale bar (whole flies) 1 mm. Scale bar (wings) 100 μm.

a CG7379 alignment with human ING1

Length: 384; Identity: 109/385 (28%); Similarity: 156/385 (41%); Gaps: 152/385 (39%)

```
CG7379          MINPIASESEAINSATYVDNYIDSVENLPDDVQRQLSRIRDIDVQYRGLIRDVDHYYDLY
ING1            MLSPANGEQ--LHLVNYVEDYMDSIESLPFDLQRNVSLMREIDAKYQEILKELDECYERF
                *:.*  .*.  :: ..**::*:**:*.** *:**::* :*:**.:*: :::::*. *: :

CG7379          LSLQNSADAGRRSRSISRMHQSLIQAQELGDEKMQIVNHMQEIIDGKLRQLDTDQQNLDL
ING1            S---RETDGAQKRRMLHCVQRALIRSQELGDEKIQIVSQMVELVENRTRQVDSHVELFEA
                    ..:*..:: * :  ::::**::*******:***.:* *:::.: **:*:. : ::

CG7379          KEDRDRYALLDDGTPSKLQRLQSPMREQGNQAGTGNGGLNGNGLLSAKDLYALGGYAGGV
ING1            QQELG-------------------------------------------------------
                ::: .

CG7379          VPGSNAMTSGNGGGSTPNSERSSHVSNGGNSGSNGNASGGGGGELQRTGSKRSRRRNESV
ING1            ---DTAGNSGKAGADRPKGEAAAQAD--------------------KPNSKRSRRQR---
                   ..* .**:.*.. *:.* :::..                    :..******:.

CG7379          VNNGSSLEMGGNESNSANEASGSGGGSGERKSSLGGASGAGQGRKASLQSASGSLASGSA
ING1            -----NNENRENASSNHDHDDGASGTPKEKK-----------------------------
                     . *   * *.. :. .*:.* . *:*

CG7379          ATSSGAAGGGGASGAGVVGGNNSGKKKKRKVRGSGASNANASTREETPPPETIDPDEPTY
ING1            --------------------AKTSKKKKR--------SKAKAEREASPADLPIDPNEPTY
                                     ::.*****        .   : ** :*.  .***:****

CG7379          CVCNQISFGEMILCDNDLCPIEWFHFSCVSLVLKPKGKWFCPNCRGERPNVMKPKAQFLK
ING1            CLCNQVSYGEMIGCDNDECPIEWFHFSCVGLNHKPKGKWYCPKCRGENEKTMDKALEKSK
                *:***:*:**** **** ***********.*  ******:**:****. :.*.   :  *

CG7379          ELERYNKEKEEKT
ING1            KERAYNR------
                : . **:
```

b CG7379 alignment with human ING2

Length: 419; Identity: 109/420 (26%); Similarity: 166/420 (40%); Gaps: 168/420 (40%)

```
CG7379          MINPIASESEAINSATYVDNYIDSVENLPDDVQRQLSRIRDIDVQYRGLIRDVDHYYDLY
ING2            --------MAIARTGVYVDDYLEYASTFPAELQRLLNTVRELDERSQSLINQTRQQTKYC
                            .:..***:*:: ...:* ::** *. :*::* : :.**.:. :  .

CG7379          LSLQNSADAGRRSRSISRMHQSLIQAQELGDEKMQIVNHMQEIIDGKLRQLDTDQQNLDL
ING2            LGLASQSSK--KGNGNHYNNGGLDEEETIEKMRKEIESSQENALSLCTEKVLLARQAYDL
                *.* ..:.   :...    : .* : : : . : :* .  :: :.   .::   :*  **

CG7379          KEDRDRYALLDDGTPSKLQRLQSPMREQGNQAGTGNGGLNGNGLLSAKDLYALGGYAGGV
ING2            IDSHVKRLDEDLNN-------------------------------FAEDLKQEG------
                 :.: :    * ..                                *:**   *

CG7379          VPGSNAMTSGNGGGSTPNSERSSHVSNGGNSGSNGNASGGGGGELQRTGSKRSRRRNESV
ING2            ------------------------------------------------------------


CG7379          VNNGSSLEMGGNESNSANEASGSGGGSGERKSSLGGASGAGQGRKASLQSASGSLASGSA
ING2            --------KIPPDEPSVLPPLPIVPKAEKRKSFYG----TPQPKKIDYRDRD--------
                            :. *.  .      : :***  *    : * :* . :. .

CG7379          ATSSGAAGGGGASGAGVVGGNNSGKKKKRKVRGSGASNANASTREETPPPETIDPDEPTY
ING2            ----------------------WDRDRDFELMPPPGSNRKDLMPIEEQP---IDPNEPTY
                                       .:.:. ::  . .** :     *  *   ***:****

CG7379          CVCNQISFGEMILCDNDLCP-IEWFHFSCVSLVLKP--KGKWFCPNCRGERPNVMKPKAQ
ING2            CVCHQVSFGDMIACDNENCQGGEWFHYTCVGLTPETRFKGKWYCPTCR------LLPQSH
                ***:*:***:** ***: *   ****::**.*. :.  ****:**.**      : *:::

CG7379          FLKELERYNKEKEEKT
ING2            ----------------
```

Fig. S3. Amino acid alignment of *Drosophila melanogaster* CG7379 with human ING1 (a) and ING2 (b). Alignment was performed using the tool on Flybase (www.flybase.org). (*) positions which have a single, fully conserved residue; (.) indicates conservation between groups of strongly similar properties; (:) indicates conservation between groups of weakly similar properties

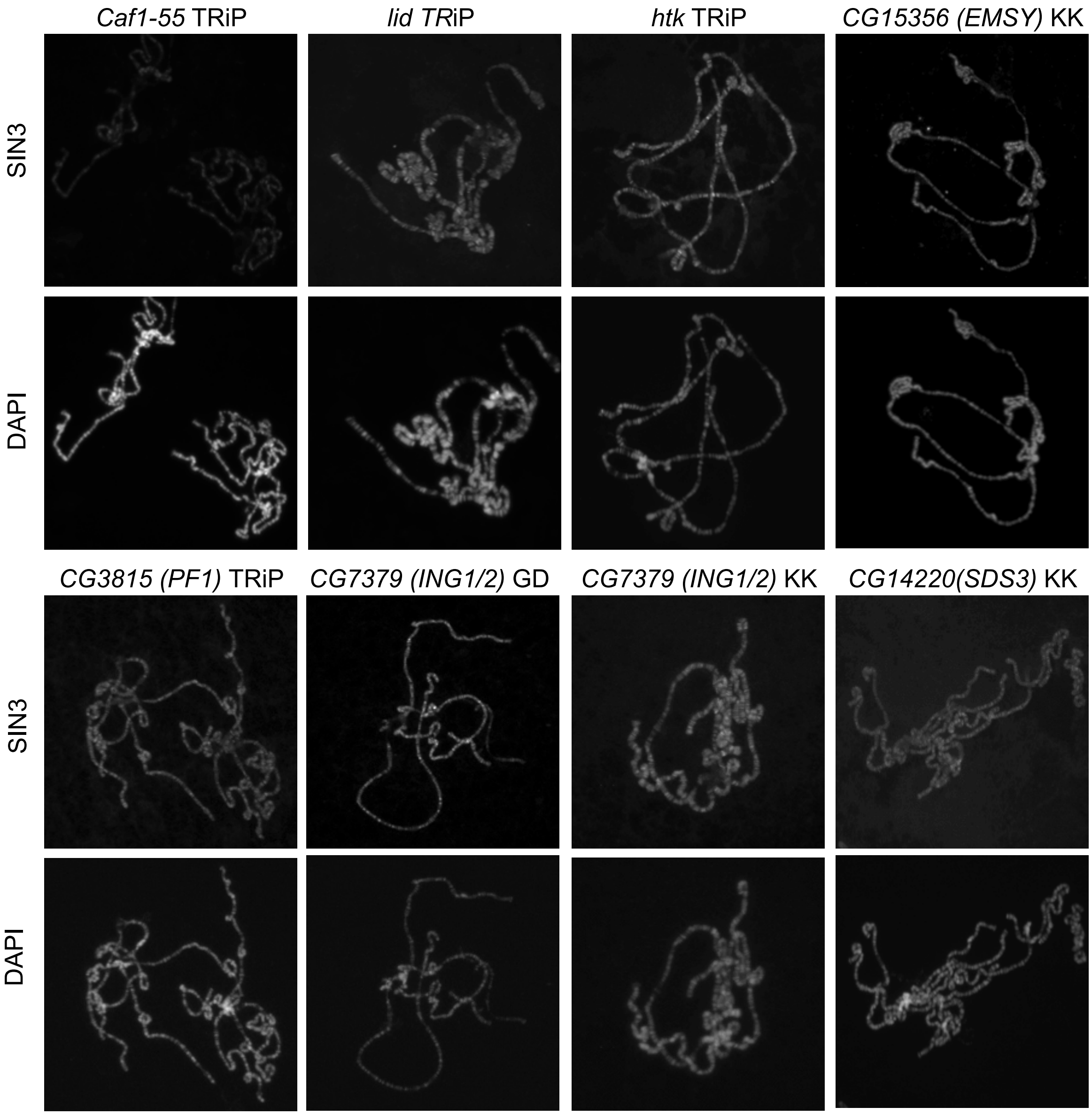

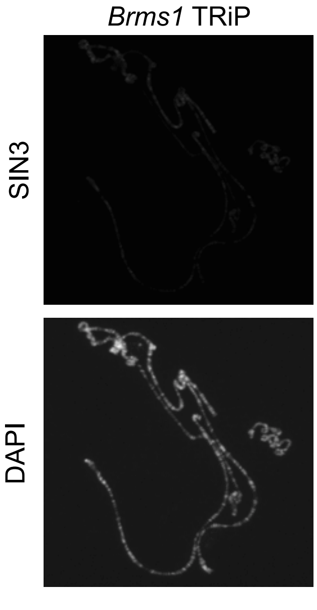

Fig. S4. Control of SIN3 chromatin recruitment by SIN3 complex factors: Additional stocks tested. Polytene chromosome spreads were prepared from salivary glands of *GFP* RNAi control and knockdown flies. Chromosomes were immunostained with antibody to SIN3 (top panels) and counterstained with DAPI (bottom). See Fig. 3b. for quantification.
